# Comparative outcomes of totally endoscopic versus direct vision mini-thoracotomy mitral valve repair: A propensity score–matched retrospective analysis

**DOI:** 10.1016/j.xjon.2026.101939

**Published:** 2026-06-23

**Authors:** Akhil Anil, Mohammad Yousuf Salmasi, Paras Dixit, Muhammed A. Mashat, Dhruvit Bhavikkumar Modi, Maged Makhoul, Toufan Bahrami

**Affiliations:** a Imperial College School of Medicine, London, United Kingdom; b Department of Cardiothoracic Surgery, Imperial College Healthcare NHS Trust, London, United Kingdom; c Department of Cardiothoracic Surgery, Royal Brompton and Harefield NHS Foundation Trust, London, United Kingdom; d Department of Surgery, King Abdulaziz University, Jeddah, Saudi Arabia; e The Harley Street Clinic, HCA Healthcare UK, London, United Kingdom

**Keywords:** minimally invasive, totally endoscopic, valve surgery, mitral valve, mitral regurgitation

## Abstract

**Background:**

Totally endoscopic mitral valve repair has emerged as an alternative minimally invasive approach to direct vision mini-thoracotomy (DVMT) repair, using 3-dimensional thoracoscopic guidance and a smaller working incision without rib spreading. Whether these technical differences translate into improved outcomes remains uncertain.

**Methods:**

A propensity score–matched, retrospective analysis was conducted on 114 patients undergoing elective mitral valve repair for degenerative mitral regurgitation (MR) via totally endoscopic or DVMT approaches at 2 UK tertiary centers between 2015 and 2024. Patients were matched 1:1 on key preoperative variables. The primary outcome was clinically significant recurrent MR on follow-up echocardiography. Secondary outcomes assessed operative parameters and recovery metrics. Multivariate regression identified predictors of relevant outcomes.

**Results:**

Totally endoscopic repair was associated with lower odds of developing clinically significant recurrent MR (odds ratio, 0.05; 95% confidence interval, 0-0.32; *P* = .0094) compared with DVMT. Intraoperative metrics were broadly comparable in the 2 groups, with the totally endoscopic procedure associated with marginal, nonsignificant increases in cardiopulmonary bypass and cross-clamp times. Immediate postrepair transesophageal echocardiography demonstrated no greater than mild residual MR in either cohort. Procedural safety was similar in the 2 groups, with no 30-day mortality and low complication rates. Trends toward shorter intensive care and postoperative hospital stays were seen in totally endoscopic patients, although these were not statistically significant.

**Conclusions:**

The totally endoscopic approach achieved more durable repairs, with comparable perioperative safety to DVMT. The observed improvement in early repair durability remains hypothesis-generating and warrants further evaluation in larger studies with standardized long-term follow-up.

**Figure:**
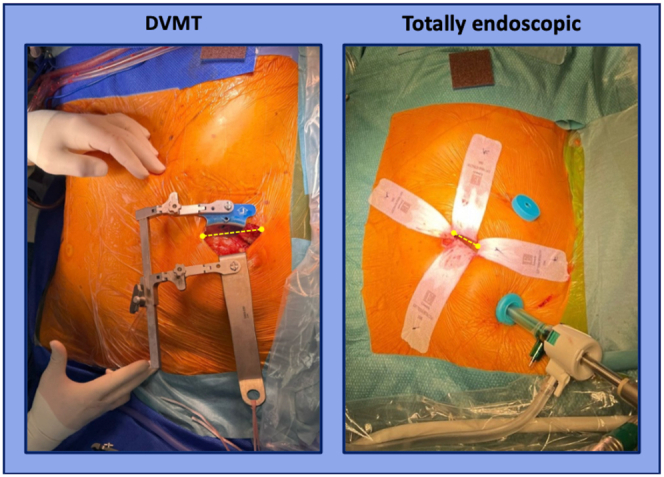
Totally endoscopic versus DVMT mitral valve repair.

**Central Message**

In our propensity score–matched cohort, totally endoscopic mitral valve repair was associated with less recurrent mitral regurgitation and comparable perioperative safety to direct vision mini-thoracotomy.

**Perspective**

In our propensity score–matched cohort, totally endoscopic mitral valve repair was associated with a lower incidence of clinically significant recurrent mitral regurgitation and comparable perioperative safety to direct vision mini-thoracotomy. Larger studies with standardized long-term echocardiographic follow-up and adjustment for surgeon experience and procedural learning curves are needed.

Mitral regurgitation (MR) is one of the most prevalent forms of valvular heart disease worldwide, accounting for a significant proportion of surgical valve interventions in modern practice.^1^ In patients with degenerative MR, mitral repair is the preferred approach, associated with fewer valve-related complications and superior long-term survival compared with valve replacement.^2^^,^^3^ With durable mitral valve repairs now routinely achievable, emphasis has been placed on reducing procedural invasiveness without compromising the quality or integrity of the valve repair.

Minimally invasive mitral surgery (MIMS) has emerged as an alternative to conventional median sternotomy, with repair performed via a direct vision mini-thoracotomy (DVMT) the most widely adopted technique.^4^ In a randomized controlled trial, no or mild MR was observed in 95% of patients at 12 weeks, compared with 96% of those who underwent sternotomy, with further evidence suggesting that valve competence is equally well preserved at long-term follow-up.^5^, ^6^, ^7^ Despite longer cardiopulmonary bypass (CPB) and aortic cross-clamp times, complication and mortality rates remained consistently low.^8^^,^^9^ Patients undergoing DVMT repairs also experienced shorter stays in both the intensive care unit (ICU) and hospital.^9^ Collectively, these findings position the right mini-thoracotomy repair as a well-validated and established standard for MIMS; however, its reliance on direct vision through a restricted intercostal incision and the need for rib spreading impose inherent limitations in visualization and minimizing surgical trauma.

In response to these limitations, totally endoscopic mitral valve repair was developed, using a smaller working incision and 3-dimensional (3D) thoracoscopic guidance. Soft tissue retractors are used to maintain a stable working space and allow the use of specialized instruments without the need for rib spreading. Previous studies have demonstrated the feasibility of this approach, with 1 study reporting that 95.1% of patients had no recurrent MR at 6-month echocardiographic follow-up.^10^ Favorable early recovery also has been reported in select cohorts, with no or minimal postoperative pain in 94% of patients and a return to normal activities within 4 weeks in one-third of cases.^11^

Although these findings are promising and support the clinical feasibility of totally endoscopic mitral valve repair, the limited availability of comparative data with the DVMT warrants further investigation. We aimed to assess the recurrence of MR on follow-up, along with intraoperative and recovery-related outcomes, to evaluate whether the technical differences of the endoscopic approach are associated with differences in repair durability and procedural safety, compared with DVMT repair.

## Methods

### Study Design and Participants

A retrospective analysis was performed on 2710 patients who underwent mitral valve surgery between January 2015 and December 2024 at either Royal Brompton or Harefield Hospital, 2 high-volume tertiary cardiac centers in the United Kingdom. Adults aged ≥18 years, who underwent elective mitral valve repair for severe, degenerative MR via a DVMT or totally endoscopic approach, were included. Concomitant procedures viable through both approaches, notably tricuspid valve repair, atrial fibrillation (AF) ablation, left atrial appendage occlusion, and patent foramen ovale closure, were permitted and did not affect eligibility for inclusion. Patients requiring urgent or emergency procedures and those undergoing repeat mitral valve surgery were excluded. Data were collected from electronic health records. The extracted variables covered baseline characteristics, operative details, preoperative and follow-up echocardiographic parameters, and recovery outcomes.

This retrospective study was approved as a clinical audit by the Research and Development Department at Royal Brompton and Harefield NHS Foundation Trust. Data were obtained from an Ethics Committee–approved valvular surgery dataset on February 1, 2025. The requirement for individual patient consent was waived.

### Outcomes

The primary outcome was the presence of clinically significant recurrent MR, defined as greater than mild in severity, as determined from the latest available follow-up echocardiogram. Secondary outcomes included intraoperative parameters, procedural safety, and postoperative recovery. Total operative duration, CPB and aortic cross-clamp times, use of an annular prosthesis, any concomitant procedures performed, conversion to sternotomy, and immediate postrepair MR severity as assessed by transesophageal echocardiography (TEE) were documented as well. Procedural safety was evaluated based on the incidence of complications, including pleural effusions, new-onset AF, pneumonia, renal failure, cerebrovascular accidents (CVA), return to theatre, and 30-day operative mortality. Postoperative recovery was assessed using length of ICU stay, total drain output, administration of blood products and postoperative hospital stay. Follow-up echocardiographic outcomes measured recurrent MR, left ventricular ejection fraction, left atrial volume index (LAVi), right ventricular dysfunction, and pulmonary artery systolic pressure (PASP).

### Operative Technique

**Figure 1:**
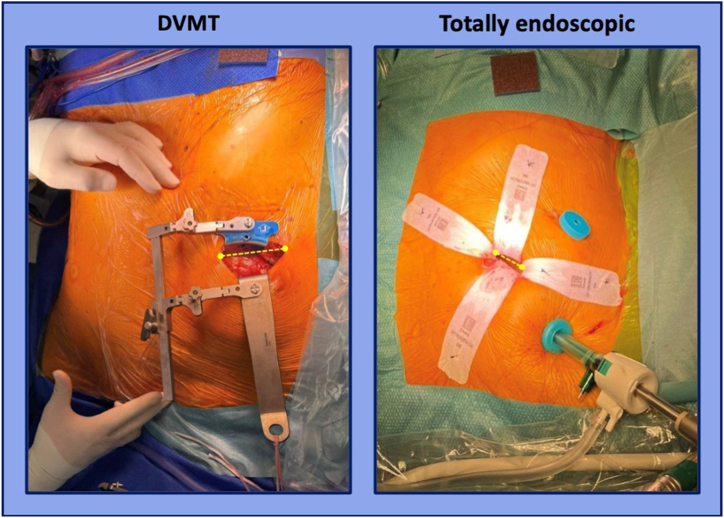
Totally endoscopic versus direct vision mini-thoracotomy (*DVMT*) mitral valve repair.

All patients underwent mitral valve repair under general anesthesia. DVMT repairs were performed through a 4- to 6-cm right anterolateral thoracotomy, whereas the totally endoscopic approach was performed using a 2- to 3-cm right anterolateral thoracotomy incision under 3D thoracoscopic guidance. CPB was established using peripheral cannulation (Figure 1). Repair techniques were consistent across both groups, with all patients undergoing ring annuloplasty. Adjunctive techniques included neochord implantation, triangular leaflet resection, and commissuroplasty, as indicated by the valve pathology.

All procedures were performed by a single surgeon in both centers. The operative technique is described in Appendix E1.

### Statistical Analysis

Statistical analysis was performed using R version 4.4.3 (R Foundation for Statistical Computing). Continuous variables were presented as mean ± standard deviation (SD) or median (interquartile range), as appropriate. Comparisons of unmatched baseline characteristics were performed using Student's t-test or Mann-Whitney U test for continuous data and χ^2^ test or Fisher's exact test for categorical variables.

Propensity scores were estimated using multivariate logistic regression, incorporating age, sex, body surface area, pulmonary disease, preoperative serum creatinine, preoperative heart rhythm, previous cardiac surgery, and ejection fraction category. Patients were then matched 1:1 using a nearest-neighbor approach with a caliper width of 0.2 SD. Matched baseline characteristics were reassessed using paired statistical tests.

Intraoperative and postoperative outcomes were analyzed using paired regression methods. Multivariate regression models were constructed to identify predictors of clinically significant recurrent MR and postoperative length of stay. Effect estimates were reported with 95% confidence intervals (CIs) and corresponding *P* values, expressed as odds ratios (ORs) for categorical outcomes and regression coefficients (β) for normally distributed continuous variables. A 2-tailed *P* value of <.05 defined statistical significance for all analyses. The complete statistical analysis is provided in Appendix E2.

## Results

### Baseline Characteristics

A total of 207 patients were included in the unmatched cohort, of whom 124 underwent DVMT mitral valve repair and 83 underwent totally endoscopic repair. In the unmatched analysis, the totally endoscopic group was significantly younger and included a lower proportion of male patients. No other statistically significant differences in baseline demographics, clinical, or echocardiographic characteristics were observed (Table 1).

**Table 1:** Baseline characteristics of patients undergoing direct vision mini-thoracotomy and totally endoscopic mitral valve repairs before and after propensity score matching

Characteristic	Unmatched			Matched		
	DVMT (N = 124)	Totally endoscopic (N = 83)	*P* value	DVMT (N = 57)	Totally endoscopic (N = 57)	*P* value
Age, y, median (IQR)∗	66.0 (18.3)	63.0 (17.0)	**.019**	63.0 (19.0)	64.0 (18.0)	.7401
Male sex, n (%)∗	91 (73)	49 (59)	**.0443**	38 (67)	35 (61)	.6276
Height, cm, mean ± SD	172.2 ± 9.7	171.9 ± 10.3	.8301	170.0 (14.0)	173.0 (15.0)	.9810
Weight, kg, median (IQR)	75.0 (21.0)	73.0 (22.7)	.5020	74.7 ± 16.0	74.0 ± 15.3	.8135
BSA, m^2^, median (IQR)∗	1.9 (0.3)	1.9 (0.4)	.5739	1.9 ± 0.2	1.9 ± 0.2	.8407
Diabetes, n (%)	5 (4)	2 (2)	.7044	0	2 (4)	.4795
Hypertension, n (%)	62 (50)	41 (50)	1.0000	25 (44)	30 (53)	.4414
Hyperlipidemia, n (%)	52 (42)	32 (39)	.7330	23 (40)	24 (42)	1.0000
Pulmonary disease, n (%)∗	5 (4)	1 (1)	.4051	2 (4)	1 (2)	1.0000
Cerebrovascular disease, n (%)	3 (2)	3 (4)	.6857	1 (2)	3 (5)	.6171
Extracardiac arteriopathy, n (%)	3 (2)	0	.2759	1 (2)	0	1.0000
Smoking status, n (%)			.2649			.5935
Nonsmoker	94 (76)	63 (76)		45 (79)	45 (79)	
Ex-smoker	30 (24)	18 (22)		12 (21)	11 (19)	
Current smoker	0	2 (2)		0	1 (2)	
Previous cardiac surgery, n (%)∗	2 (2)	0	.5173	0	0	1.0000
Preoperative serum creatinine, μmol/L, mean ± SD	81.5 ± 17.3	79.6 ± 17.1	.4439	75.0 (19.0)	82.0 (22.0)	.7757
Preoperative heart rhythm, n (%)∗			.6362			.5428
Sinus rhythm	102 (82)	65 (78)		46 (80)	43 (75)	
Atrial fibrillation/flutter	21 (17)	16 (19)		11 (19)	13 (23)	
Other	1 (1)	2 (2)		0	1 (2)	
Ejection fraction category, n (%)∗			.4412			.9311
Good (>50%)	112 (90)	76 (92)		53 (93)	54 (95)	
Moderate (31%-50%)	12 (10)	6 (7)		4 (7)	3 (5)	
Poor (<31%)	0	1 (1)		0	0	
NYHA classification, n (%)			.9164			.9845
I	10 (8)	7 (8)		3 (5)	3 (5)	
II	55 (44)	38 (46)		26 (46)	25 (44)	
III	55 (44)	34 (41)		25 (44)	25 (44)	
IV	4 (3)	4 (5)		3 (5)	4 (7)	

Bold type indicates statistical significance. *IQR*, Interquartile range; *BSA*, body surface area; *DVMT*, direct vision mini-thoracotomy; *NYHA*, New York Heart Association.

∗ Propensity score–matched covariates.

**Figure E1:**
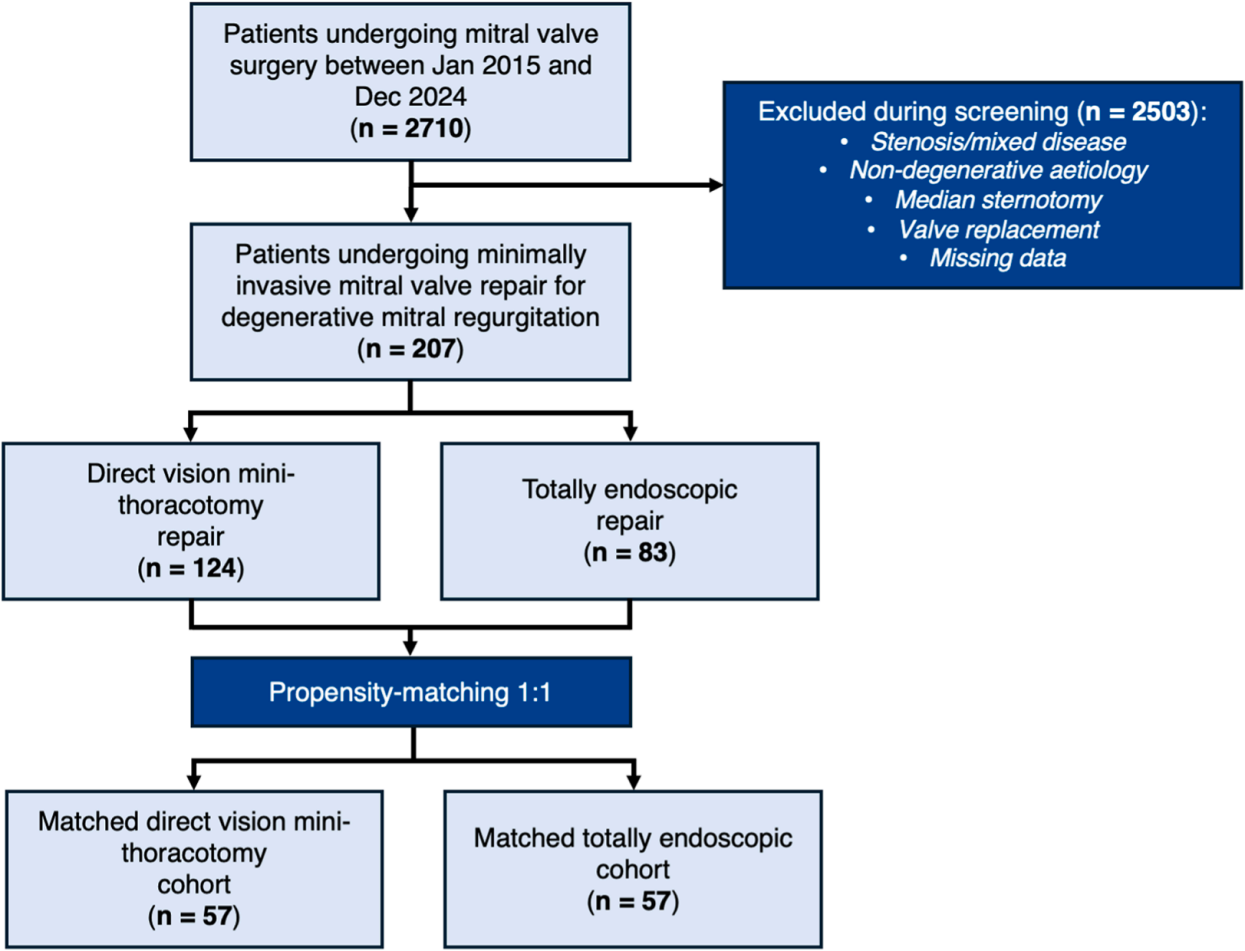
Flow diagram illustrating patient selection and formation of propensity score–matched patient cohorts.

After propensity score matching, 57 patients were retained in each group (Figure E1). In these patients, 64 procedures were performed at Harefield Hospital (33 DVMT, 31 totally endoscopic) and 50 were performed at Royal Brompton Hospital (24 DVMT, 26 totally endoscopic). Reassessment of baseline characteristics demonstrated adequate balance across all matched covariates, with no residual imbalances in other clinically relevant variables, including preoperative heart rhythm, New York Heart Association class, and key comorbidities.

### Preoperative TEE Findings

Preoperative TEE findings are summarized in Table 2. Mitral valve pathology characteristics were similar in the 2 groups, with posterior leaflet prolapse the most frequently observed lesion. Left ventricular systolic function, LAVi, right ventricular systolic dysfunction, and PASP were comparable between the groups. Overall, no statistically significant between-group differences were seen during preoperative echocardiographic assessment.

**Table 2:** Preoperative transesophageal echocardiographic findings

Parameter	DVMT (N = 57)	Totally endoscopic (N = 57)	*P* value
Mitral valve pathology, n (%)			.3438
Anterior leaflet prolapse	5 (9)	3 (5)	
Posterior leaflet prolapse	38 (67)	37 (65)	
Bileaflet prolapse	12 (21)	17 (30)	
Other	2 (4)	0	
LVEF, %, mean ± SD	63.1 ± 7.9	61.7 ± 7.8	.2665
LA volume index (mL/m^2^), median (IQR)∗	56.5 (26.9)	57.4 (24.1)	.8919
Right ventricular systolic dysfunction, n (%)†			.6077
None	47 (86)	51 (93)	
Mild	5 (9)	3 (6)	
Moderate	2 (4)	1 (2)	
Severe	1 (2)	0	
PASP, mm Hg, median (IQR)‡	35.5 (23.5)	31.0 (7.8)	.0575

Comparisons are based on 1:1 propensity score–matched cohorts (n = 57 per group); sample sizes vary for some variables because of missing data. *DVMT*, Direct vision mini-thoracotomy; *LVEF*, left ventricular ejection fraction; *LA*, left atrium; *IQR*, interquartile range; *PASP*, pulmonary artery systolic pressure.

∗ 51 matched pairs.

† 55 matched pairs.

‡ 22 matched pairs.

### Intraoperative Parameters

Intraoperative parameters are reported in Table 3. Concomitant procedures, including AF ablation, left atrial appendage occlusion, and patent foramen ovale closure, were performed in both groups. The mean total operative time was similar in the DVMT group (336.3 ± 101.0 minutes) and the totally endoscopic group (337.0 ± 66.4 minutes). Mean CPB time was longer in the totally endoscopic group (161.4 ± 52.3 minutes vs 171.5 ± 38.1 minutes), with a corresponding trend toward longer aortic cross-clamp times (94.0 ± 31.0 minutes vs 104.0 ± 39 minutes); however, neither difference reached statistical significance. No patients were converted to a sternotomy.

**Table 3:** Intraoperative parameters

Parameter	DVMT (N = 57)	Totally endoscopic (N = 57)	Effect estimate (95% CI)	*P* value
Concomitant procedures, n (%)∗	15 (26%)	19 (33%)	1.57 (0.61-4.05)	.3499
LAA occlusion	7 (12%)	15 (26%)	5.00 (1.10-22.82)	**.03773**
AF ablation	7 (12%)	10 (18%)	1.50 (0.53-4.21)	.4417
PFO closure	3 (5%)	5 (9%)	1.67 (0.40-6.97)	.4843
Total operative time, min	336.3 ± 101.0	337.0 ± 66.4	0.72 (−28.47 to 29.91)	.9617
CPB time, min	161.4 ± 52.3	171.5 ± 38.1	10.16 (−4.16 to 24.48)	.1699
Aortic cross-clamp time, min†	94.0 (31.0)	104.0 (39.0)	1.10 (1.00-1.22)	.0520
Conversion to sternotomy, n	0	0		1.0000
Postrepair MR, n (%)			0.20 (0.02-1.71)	.1418
None	52 (91%)	56 (98%)		
Mild	5 (9%)	1 (2%)		
Moderate	0	0		
Severe	0	0		

Comparisons are based on 1:1 propensity score–matched cohorts (n = 57 per group). Effect estimates are reported as odds ratios for binary outcomes, as regression coefficients for normally distributed continuous variables, and as geometric mean ratios for log-transformed variables. Bold type indicates statistical significance. *DVMT*, Direct vision mini-thoracotomy; *CI*, confidence interval; *LAA*, left atrial appendage; *AF*, atrial fibrillation; *PFO*, patent foramen ovale; *MR*, mitral regurgitation.

∗ Patients may have received 1 or more concomitant procedures.

† Log-transformed variable.

Immediate postrepair TEE demonstrated satisfactory valve competence in both cohorts. No patient had greater than mild residual MR, with comparable postrepair MR severity in the 2 groups.

### Early Postoperative Recovery Metrics

Early postoperative recovery metrics are shown in Table 4. The median ICU stay was shorter following totally endoscopic repair (40.5 hours vs 25.6 hours), but the difference was not statistically significant. No significant between-group differences were observed in blood transfusion requirements, total drain output, or rate of return to the operating theatre.

**Table 4:** Early postoperative recovery metrics

Parameter	DVMT (N = 57)	Totally endoscopic (N = 57)	Effect estimate (95% CI)	*P* value
ICU stay, h, median (IQR)∗	40.5 (26.3)	25.6 (24.9)	0.88 (0.72-1.09)	.2433
Blood transfused, n (%)	27 (48)	27 (48)	1.00 (0.50-2.00)	1.0000
Total drain output, mL, median (IQR)∗	1250.0 (1250.0)	1325.0 (955.0)	1.12 (0.89-1.40)	.3404
Return to theatre, n (%)	4 (7)	3 (5)	0.75 (0.17-3.35)	.7064
New-onset AF, n (%)	1 (2)	3 (5)	3.00 (0.31-28.84)	.3414
Pleural effusion, n (%)			1.53 (0.70-3.31)	.2855
None	41 (72)	35 (61)		
Small, not requiring drainage	11 (19)	17 (30)		
Large, requiring drainage	5 (9)	5 (9)		
Pneumonia, n (%)	10 (18)	6 (11)	0.56 (0.19-1.66)	.2920
Renal failure, n	0	0		1.0000
CVA, n (%)	1 (2)	0		.9993
Postoperative hospital stay, d, median (IQR)∗	7.0 (7.0)	6.0 (4.6)	0.91 (0.76-1.09)	.2926
30-d operative mortality, n	0	0		1.0000

Comparisons are based on 1:1 propensity score–matched cohorts (n = 57 per group). Effect estimates are reported as odds ratios for binary outcomes, as regression coefficients for normally distributed continuous variables, and as geometric mean ratios for log-transformed variables. *DVMT*, Direct vision mini-thoracotomy; *CI*, confidence interval; *ICU*, intensive care unit; *IQR*, interquartile range; *AF*, atrial fibrillation; *CVA*, cerebrovascular accident.

∗ Log-transformed variable.

Postoperative complications, including new-onset AF, pleural effusion, pneumonia, renal failure, and CVA, were infrequent and comparable in the 2 groups. The median postoperative hospital stay was marginally shorter in the totally endoscopic group, but the difference was not statistically significant (7.0 days vs 6.0 days). No deaths occurred within 30 days of surgery.

### Follow-up Echocardiographic Outcomes

Follow-up echocardiograms were performed between 3 and 12 months postoperatively; results are reported in Table 5. Clinically significant recurrent MR (moderate or severe) occurred more frequently following DVMT repair than after totally endoscopic repair (21% vs 4%), with significantly lower odds of overall recurrence in the totally endoscopic group (OR, 0.38; 95% CI, 0.18-0.82; *P* = .0142).

**Table 5:** Follow-up echocardiographic outcomes

Outcome	DVMT (N = 57)	Totally endoscopic (N = 57)	Effect estimate (95% CI)	*P* value
Recurrent mitral regurgitation, n (%)			0.38 (0.18-0.82)	**.0142**
None	15 (26)	23 (40)		
Mild	30 (53)	32 (56)		
Moderate	7 (12)	1 (2)		
Severe	5 (9)	1 (2)		
LVEF, %, median (IQR)	55.0 (10.0)	55.0 (11.0)	0.98 (0.92-1.06)	.6694
LA volume index, mL/m^2^, median (IQR)∗,†	38.5 (10.1)	37.8 (23.7)	1.00 (0.85-1.17)	.9935
RV systolic dysfunction, n (%)			0.56 (0.26-1.20)	.1342
None	32 (56)	40 (70)		
Mild	21 (37)	14 (25)		
Moderate	3 (5)	2 (4)		
Severe	1 (2)	1 (2)		
PASP, mm Hg, median (IQR)∗,‡	25.0 (8.8)	22.0 (10.3)	0.80 (0.64-1.01)	.0792

Comparisons are based on 1:1 propensity score–matched cohorts (n = 57 per group); sample sizes vary for certain variables because of missing data. Effect estimates are reported as odds ratios for binary outcomes, as regression coefficients for normally distributed continuous variables, and as geometric mean ratios for log-transformed variables. Bold type indicates statistical significance. *DVMT*, Direct vision mini-thoracotomy; *CI*, confidence interval; *LVEF*, left ventricular ejection fraction; *IQR*, interquartile range; *LA*, left atrium; *RV*, right ventricular; *PASP*, pulmonary artery systolic pressure.

∗ Log-transformed variable.

† 50 matched pairs.

‡ 18 matched pairs.

Left ventricular function, LAVi, and right ventricular systolic function remained similar in the 2 groups. A trend toward a lower PASP was observed in the totally endoscopic group, but this did not reach statistical significance.

### Multivariate Regression Analyses

**Figure E2:**
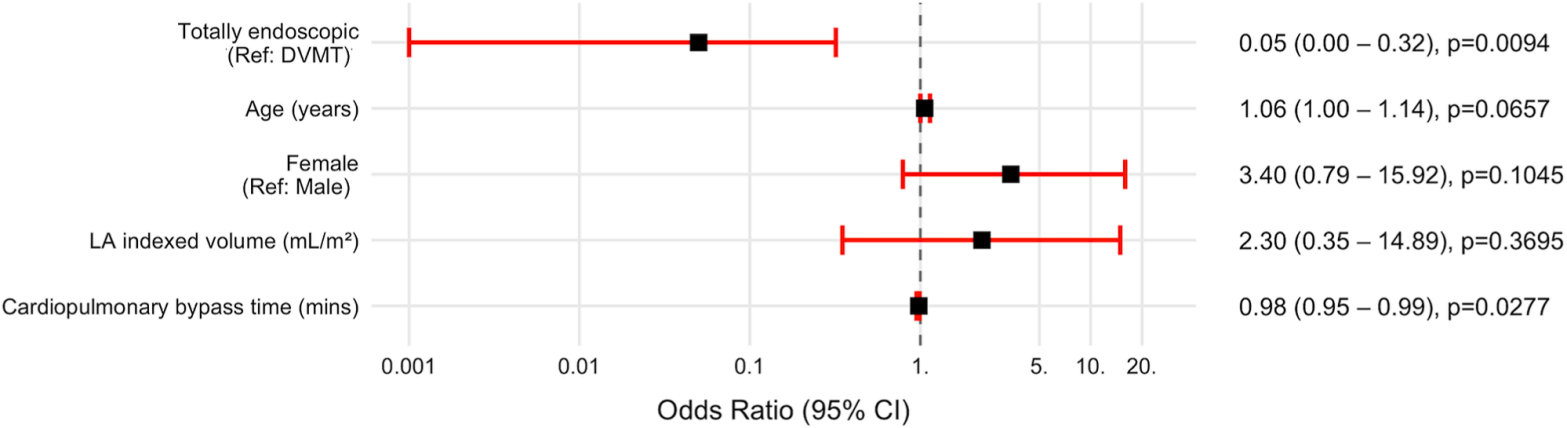
Forest plot depicting the multivariate logistic regression model for clinically significant recurrent mitral regurgitation. The plot shows multivariate odds ratios (*ORs*) with 95% confidence intervals (*CIs*) and corresponding *P* values. The *black squares* represent point estimates, and the *horizontal red lines* indicate 95% CI. The *vertical dashed line* denotes the line of no effect (OR = 1). Exact ORs, 95% CIs, and *P* values are displayed in the right panel. The *x*-axis is shown on a logarithmic scale. *DVMT*, Direct vision mini-thoracotomy; *LA*, left atrium.

Multivariate logistic regression analysis is shown in Table E1 and Figure E2, identifying independent predictors of clinically significant MR recurrence. Totally endoscopic repairs were independently associated with lower odds of clinically significant recurrent MR compared with DVMT repairs (OR, 0.05; 95% CI, 0-0.32; *P* = .0094).

CPB time demonstrated a modest but statistically significant effect, with longer durations linked to lower odds of clinically significant recurrent MR. Older age was associated with a borderline increase in the odds of developing clinically significant recurrent MR. Sex and LAVi did not show a significant association in the adjusted model.

**Figure E3:**
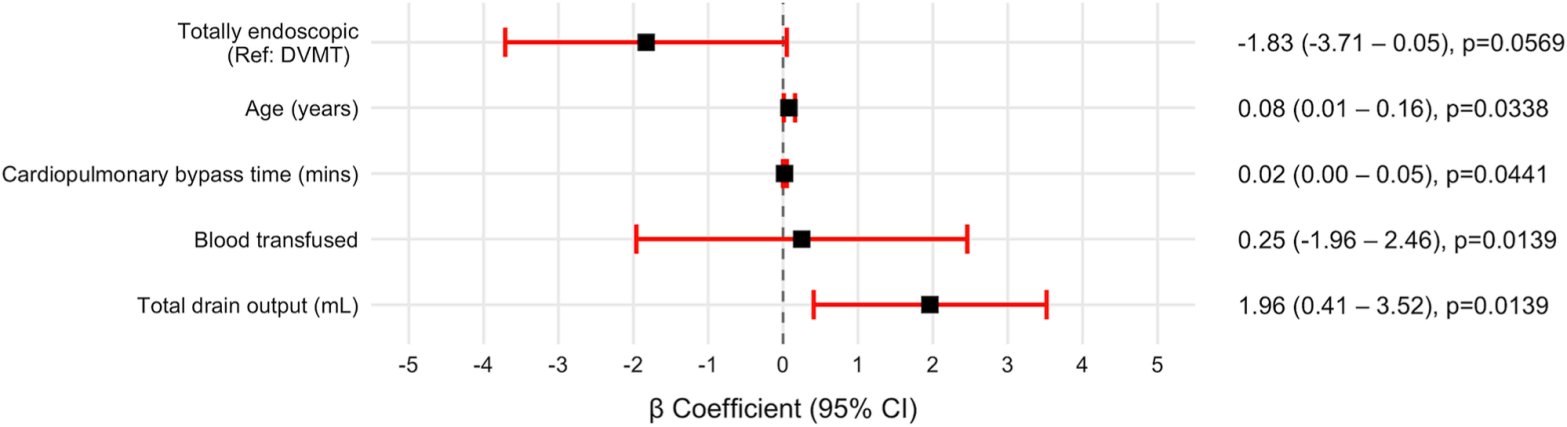
Forest plot depicting the multivariate linear regression model for postoperative hospital length of stay. The plot shows multivariate regression coefficients (*β*) with 95% confidence intervals (*CIs*) and corresponding *P* values. The *black squares* represent point estimates, with *horizontal red lines* indicating 95% CIs. The *vertical dashed line* denotes the line of no effect (β = 0). Exact regression coefficients, 95% CIs, and *P* values are displayed in the right panel. *DVMT*, Direct vision mini-thoracotomy.

Multivariate linear regression results for postoperative hospital length of stay are shown in Table E2 and Figure E3. Surgical approach was not independently associated with length of stay, although a trend toward shorter stays following the endoscopic approach was observed (β = −1.83; 95% CI, −3.71 to 0.05; *P* = .0569).

Older age, longer CPB time, blood transfusion requirement, and total drain output were independently associated with longer postoperative hospital stay.

## Discussion

This propensity score–matched retrospective study is among the first to directly compare the totally endoscopic and DVMT approaches for mitral valve repair. The principal finding was a significantly lower incidence of recurrent MR following totally endoscopic repair. Importantly, immediate post-repair TEE demonstrated satisfactory valve competence in both groups, with no patient leaving the operating theatre with greater than mild residual MR. Perioperative safety was comparable, with low rates of postoperative complications and no 30-day mortality, despite modestly longer CPB and cross-clamp times. Although not statistically significant, totally endoscopic patients also showed a slight trend toward shorter ICU and postoperative length of stay. These findings suggest a potential difference in early repair durability between the 2 approaches but should be interpreted cautiously given the retrospective design, short follow-up interval, single-surgeon experience, and temporal transition between techniques.

### Intraoperative Outcomes

Intraoperative metrics were broadly comparable between the approaches, with nonsignificant increases in CPB and cross-clamp times observed in the totally endoscopic group. These differences may reflect the greater technical complexity of the technique, with the smaller working incision requiring coaxial hand–instrument alignment. Unlike DVMT, in which direct visualization and rib spreading offer a more flexible approach angle, the repair is performed along a restricted trajectory under thoracoscopic guidance. This is consistent with prior comparisons between minimally invasive and sternotomy approaches, where longer operative times have been attributed to restricted instrument mobility.^12^ Although this setup demands more measured, time-intensive maneuvers, immediate postrepair TEE demonstrated successful repairs, with 98% of patients having no residual MR. However, given the limited comparative evidence, it is difficult to determine whether the differences are solely a result of the procedure's technical demands or arise from residual confounding due to unmeasured factors.

### Early Postoperative Recovery Metrics

Early postoperative complications were infrequent and comparable between the 2 groups. There were no significant differences in rates of return to theatre, pleural effusions, new-onset AF, pneumonia, renal failure, or CVA, and no 30-day operative mortality was observed in either group. Although the totally endoscopic approach eliminates rib spreading and reduces the size of the working incision, both techniques remain within the spectrum of MIMS.^13^ In this setting, the marginal difference in invasiveness might not be sufficient to yield meaningful variation in postoperative morbidity, with complications more likely driven by patient-specific risk factors and intraoperative decision making than by access strategy alone.^14^^,^^15^

Patients undergoing totally endoscopic repair demonstrated modest reductions in ICU and postoperative hospital length of stay. Although these differences did not reach statistical significance, their direction aligns with the expected benefits of a non–rib-spreading technique and still may be clinically relevant and merits further evaluation in larger cohorts.^16^

### Follow-up Echocardiographic Outcomes

On follow-up echocardiography, totally endoscopic repair was associated with substantially lower odds of developing clinically significant recurrent MR compared to DVMT repair. Notably, all patients who developed moderate or severe MR at follow-up had no greater than mild residual MR on immediate postrepair TEE, suggesting that the recurrent MR was unlikely to reflect residual regurgitation at the time of repair. While causality cannot be established, one possible explanation for this is the different visualization strategies. Mini-thoracotomies rely on direct vision through a 4- to 6-cm incision, which may limit the operative field, making it challenging to assess the mitral apparatus as a whole.^17^ Suboptimal lighting may further impair visibility, particularly subvalvular structures such as the chordae tendineae and papillary muscles.^18^ Exposure also relies on manual soft tissue retraction and frequent instrument repositioning, which may result in variable visualization, particularly in patients with unfavorable chest wall anatomy.^13^^,^^19^

In contrast, the totally endoscopic technique uses high-definition 3D thoracoscopic imaging, providing a stable, magnified panoramic view of the mitral apparatus.^20^ This may allow for a more detailed, shared assessment of the leaflet morphology, commissural symmetry, and subvalvular anatomy. The fixed camera position ensures a consistent operative view for all members of the surgical team, eliminating the variability introduced by changes in surgeon positioning or retractor placement. Preserved depth perception may also assist with technically demanding aspects of the repair, including neochord placement and annuloplasty ring sizing.^16^^,^^21^ Together, these features may provide a plausible explanation for the lower incidence of recurrent MR after totally endoscopic repair; however, this mechanism remains speculative and cannot be confirmed from this study.

Multivariate analysis also identified longer CPB time as an independent predictor of reduced MR recurrence; however, this association is most likely confounded by collinearity, as totally endoscopic cases tended to have both modestly longer CPB times and lower rates of MR recurrence. As such, this finding is unlikely to reflect a true protective effect with prolonged CPB.

### Limitations and Future Directions

This study has several limitations. Its retrospective, nonrandomized design introduces the possibility of selection bias and residual confounding despite propensity matching. The matched cohort was small, with 57 patients in each group, limiting statistical power and the precision of effect estimates.

The selection of surgical approach reflected evolving institutional practice within a single-surgeon experience. DVMT repairs were performed earlier in the study period, followed by a gradual transition toward the totally endoscopic approach. Although this limits intersurgeon variability, it makes it difficult to separate the effect of surgical approach from increasing repair experience over time, particularly as procedural learning curves were not adjusted for. This temporal separation also may have introduced confounding owing to changes in care independent of surgical technique, such as anesthetic practices, recovery protocols, and ICU management. Importantly, the generalizability of these findings should be considered in the context of surgeon experience and institutional expertise. Safe adoption of the technique is likely dependent on careful case selection, established mitral expertise, dedicated minimally invasive infrastructure, and program-level support.

The lack of a standardized echocardiographic follow-up protocol limits the precision with which valve durability can be assessed in both techniques.^22^ Although all patients underwent follow-up assessment between 3 and 12 months, this interval remains short and variable for evaluating repair durability. Longer-term follow-up is needed to determine whether the observed difference in recurrent MR persists over time. Future studies should prioritize larger multicenter cohorts with standardized long-term follow-up and adjustment for surgeon experience, along with incorporation of patient-reported outcome measures (visual analog scale and Short Form-36).^23^^,^^24^

## Conclusions

In conclusion, totally endoscopic mitral valve repair appears to provide a safe and effective alternative to the DVMT repair, with a trend toward improved early repair durability. Rather than establishing the inherent superiority of one approach, this study highlights the potential importance of operative visualization, technical execution, and surgeon experience in determining surgical outcomes. The observed reduction in recurrent MR should be viewed as a promising but hypothesis-generating finding that requires validation in larger, prospective studies with standardized long-term echocardiographic follow-up before broader conclusions can be drawn about its role in standard surgical practice.

## Conflict of Interest Statement

The authors reported no conflicts of interest.

The *Journal* policy requires editors and reviewers to disclose conflicts of interest and to decline handling or reviewing manuscripts for which they may have a conflict of interest. The editors and reviewers of this article have no conflicts of interest.

## Figures and Tables

**Table E1:** Multivariate logistic regression model for clinically significant recurrent MR

Variable	Reference category	Univariate analysis		Multivariate analysis	
		OR (95% CI)	*P* value	OR (95% CI)	*P* value
Surgical approach: totally endoscopic	DVMT	0.14 (0.02-0.53)	.**0116**	0.05 (0-0.32)	**.0094**
Age (y)		1.04 (0.99-1.10)	.1239	1.06 (1.00-1.14)	.0657
Female sex	Male	2.71 (0.87-8.84)	.0862	3.40 (0.79-15.92)	.1045
LA indexed volume (mL/m^2^)∗		2.36 (0.48-10.96)	.2698	2.30 (0.35-14.89)	.3695
CPB time (min)		0.98 (0.96-0.99)	.**0094**	0.98 (0.95-0.99)	**.0277**

Univariate and multivariate odds ratios (ORs) with 95% confidence intervals (CIs) and corresponding *P* values are presented. The ORs for log-transformed variables represent the multiplicative change in odds for a 1 unit increase. Bold type indicates statistical significance. *MR*, Mitral regurgitation; *OR*, odds ratio; *CI*, confidence interval; *DVMT*, direct vision mini-thoracotomy; *LA*, left atrium; *CPB*, cardiopulmonary bypass.

∗ Log-transformed variable.

**Table E2:** Multivariate linear regression model for postoperative hospital length of stay

Variable	Reference category	Univariate analysis		Multivariate analysis	
		β (95% CI)	*P* value	β (95% CI)	*P* value
Surgical approach: totally endoscopic	DVMT	−1.41 (−3.49 to 0.67)	.1823	−1.83 (−3.71 to 0.05)	.0569
Age (y)		0.41 (0.06-0.21)	**.0005**	0.08 (0.01-0.16)	**.0338**
CPB (min)		0.04 (0.02-0.06)	**.0009**	0.02 (0-0.05)	**.0441**
Blood transfused		2.99 (0.97-5.02)	**.0041**	0.25 (−1.96 to 2.46)	**.0139**
Total drain output (mL)∗		2.93 (1.57-4.30)	**<.0001**	1.96 (0.41-3.52)	**.0139**

Univariate and multivariate regression coefficients with 95% CIs and corresponding *P* values are presented. The regression coefficients for log-transformed variables represent the multiplicative change in the outcome for a one-unit increase. Bold type indicates statistical significance. *CI*, Confidence interval; *DVMT*, direct vision mini-thoracotomy; *CPB*, cardiopulmonary bypass.

∗ Log-transformed variable.

## Appendix E1 Detailed Operative Techniques for Totally Endoscopic and Direct Vision Mini-Thoracotomy Mitral Valve Repairs

The patients received general anesthesia and were intubated with a left double-lumen endotracheal tube to facilitate single-lung ventilation, allowing deflation of the right lung for optimal exposure. They were then positioned supine, with the right hemithorax elevated 30° and the right arm abducted and secured below the posterior axillary line. For the DVMT approach, a 4- to 6-cm incision was made in the fourth intercostal space along the right anterior axillary line, and a rib-spreading retractor was used to facilitate exposure. In contrast, for the totally endoscopic procedure, a 2- to 3-cm subareolar or inframammary incision was made, and soft tissue retractors were used. A separate 5- to 10-mm port was placed in the mid-axillary line for thoracoscopic guidance in the endoscopic approach.

Heparin was then given to achieve an activated clotting time >480 seconds. For both techniques, CPB was established peripherally via the femoral vessels. A 2- to 3-cm groin incision was used to expose the vessels, followed by cannulation using the Seldinger technique. The arterial cannula was advanced to the descending aorta. Bicaval venous drainage was achieved through the femoral vein to the inferior vena cava and through the internal jugular vein to the superior vena cava. Next, the aorta was cross-clamped using a Chitwood clamp, and antegrade cold blood cardioplegia was administered to the proximal ascending aorta to arrest the heart. The mitral valve was then exposed through a left atriotomy along Sondergaard's groove, and a left atrial retractor was inserted.

Repair techniques were consistent across the 2 groups, with all patients undergoing ring annuloplasty using an annular prosthesis. Adjunctive techniques included neochord implantation, triangular leaflet resection and commissuroplasty, as indicated by the valve pathology. Following the repair, the left atriotomy was closed, and deairing was performed under TEE guidance prior to removing the aortic cross-clamp. Valve competence was confirmed using TEE, after which the patients were weaned off CPB. Decannulation was performed, and hemostasis was controlled using manual compression.

## Appendix E2 Statistical Analysis and Model Specification

Statistical analysis was performed using R version 4.4.3. Continuous variables were assessed for normality using the Shapiro-Wilk test. Normally distributed data are presented as mean ± SD; non-normally distributed data, as median (interquartile range). Unmatched baseline characteristics were compared using the Student *t**-*test for parametric and the Mann-Whitney *U* test for nonparametric data. Categorical variables were analyzed using the χ^2^ test or Fisher exact test, with the latter applied when any expected frequency was <5.

Propensity scores were estimated by means of multivariate logistic regression, incorporating age, sex, body surface area, pulmonary disease, preoperative serum creatinine, preoperative heart rhythm, previous cardiac surgery, and ejection fraction category. Patients were then matched 1:1 using a nearest-neighbor approach with a caliper width of 0.2 SD. Matched baseline characteristics were reassessed using the paired *t**-*test for parametric variables, Wilcoxon signed-rank test for nonparametric variables, McNemar test for binary outcomes, and Stuart-Maxwell test for multilevel categorical outcomes.

Intraoperative and postoperative outcomes were analyzed using paired regression methods. Binary outcomes were modeled using conditional logistic regression, ordinal outcomes using cumulative link mixed models, and continuous outcomes using linear mixed-effects models. Multivariate regression models were constructed to identify predictors of clinically significant recurrent MR and postoperative length of stay. Results from univariate logistic and linear regression analyses informed variable selection. Effect estimates were reported with 95% CIs and corresponding *P* values, expressed as ORs for categorical outcomes and regression coefficients (β) for normally distributed continuous variables. Log transformation was applied to continuous data where necessary to satisfy assumptions of normality and homoscedasticity. Results from these models were exponentiated and reported as geometric mean ratios. A 2-tailed *P* value of <.05 defined statistical significance for all analyses.
